# Global Frequency and Clinical Features of Stroke in Patients With Tuberculous Meningitis: A Systematic Review

**DOI:** 10.1001/jamanetworkopen.2022.29282

**Published:** 2022-09-01

**Authors:** Marie Charmaine C. Sy, Adrian I. Espiritu, Jose Leonard R. Pascual

**Affiliations:** 1 Division of Adult Neurology, Department of Neurosciences, College of Medicine and Philippine General Hospital, University of the Philippines Manila, Manila, Philippines; 2 Department of Clinical Epidemiology, College of Medicine, University of the Philippines Manila, Manila, Philippines

## Abstract

**Importance:**

Stroke in tuberculous meningitis (TBM) is associated with significant morbidity and mortality.

**Objective:**

To determine the country-specific, regional, and overall prevalence of stroke among patients with TBM, including their clinical manifestations, stroke locations, and outcomes.

**Evidence Review:**

This systematic review searched records in MEDLINE by PubMed, Scopus, and EMBASE until July 2020 for relevant articles on the occurrence and characteristics of stroke in TBM. Randomized clinical trials and cohort studies that included a population of patients with TBM were analyzed for clinical manifestations, type of stroke, area of stroke, vascular territory, and outcomes. Studies that did not report the occurrence of stroke, reported as abstract only with no full-texts available, and articles not in English were excluded. The country-specific, regional, and overall frequencies of stroke among patients with TBM were determined; secondary analysis enumerated the summary estimates of the clinical presentations, common locations of stroke, and outcomes. The Murad tool was used to assess methodological quality.

**Findings:**

From 852 articles identified, 71 studies involving 2194 patients with stroke in TBM were included. The sample size for each study ranged from 17 to 806 patients. The frequency of stroke in TBM showed an estimate of 0.30 (95% CI, 0.26-0.33). The most common clinical manifestations were fever and headache. The lateral striate, middle cerebral, and medial striate arteries were typically affected. The basal ganglia, cortex and lobar, and internal capsule were the frequently involved areas of the brain. The pooled proportions of mortality and poor outcomes were 0.22 (95% CI, 0.16-0.29) and 0.51 (95% CI, 0.37-0.66), respectively.

**Conclusions and Relevance:**

The results of this systematic review suggest that stroke is considerably frequent among patients with TBM. The reported frequencies of stroke in TBM and its clinical features vary across the studies and populations.

## Key Points

**Question:**

What is the global frequency of stroke in patients with tuberculous meningitis (TBM)?

**Findings:**

In this systematic review including 852 articles involving 2194 patients with stroke in TBM, the estimated frequency of stroke in TBM was 0.30 (95% CI, 0.26-0.33), and ranged from 0.08 in Saudi Arabia to 0.56 in France.

**Meaning:**

These results suggest that global trends of stroke occurrence in TBM may have regional or country-specific characteristics that may be associated with disability and mortality in these patients.

## Teaser

This systematic review examines the country-specific, regional, and overall prevalence of stroke experienced by patients with tuberculous meningitis.

## Introduction

Tuberculosis (TB) is a serious infectious disease that causes high morbidity and mortality especially in developing countries.^1^ TB typically affects the lungs, but the pathogen can travel through the bloodstream and damage any organ in the body. *Mycobacterium tuberculosis* is the most common organism causing tuberculous infection in the central nervous system reaching 10% to 15% prevalence among all extrapulmonary manifestations.^2,3^

Tuberculous meningitis (TBM) accounts for 1% to 2% of cases of active TB and may lead to severe neurologic disability.^4^ The spectrum of complications of TBM include hydrocephalus, tuberculoma formation, and stroke.^3,4^ Stroke among patients with TBM can cause irreversible brain damage and lead to poor clinical outcomes.^5^ The pathophysiologic mechanism responsible for cerebral infarction include obliterative vasculitis, intimal proliferation, and hypercoagulable state.^3,6^

Currently, the reported frequencies of stroke in TBM and its clinical features seem to vary across studies conducted in different countries. Thus, the purpose of this study was to determine the country-specific, regional, and overall frequencies of stroke among patients with TBM. We also aimed to determine the summary estimates of the clinical presentations, common locations of stroke, and outcomes of these patients.

## Methods

This review complied with the Preferred Reporting Items for Systematic reviews and Meta-analyses (PRISMA) reporting guideline for systematic reviews.^7^ The protocol was registered in PROSPERO (CRD42020209704).

### Criteria for Selection of Studies

We considered randomized controlled trials and cohort studies that included a population of patients diagnosed with TBM and reported frequencies of stroke. We included studies that reported information on clinical manifestations, type of stroke, area of stroke, vascular territory, and outcomes. We considered the following outcomes for this review: mortality, recovered, and poor outcome. In this review, mortality was defined as patients with TBM who have died from the complications of TBM; recovery was implied in the studies by discharge from the hospital; and poor outcome was defined as having a modified Rankin scale score of 3 to 6. There were no restrictions implemented in terms of age, sex, and ethnicity of the population. We excluded studies that did not report the occurrence of stroke in TBM, reported as abstract only with no full-texts available and articles not in English. The Murad tool was used to evaluate the risk of bias in noncomparative cohorts.^8^ We considered poor, moderate, or good quality when 3 or fewer, 4, or 5 of the 5 criteria were fulfilled, respectively. The investigators evaluated the methodological quality and resolved the discrepancies of the included studies.

### Search Methods for the Identification of Studies and Study Selection

We conducted a comprehensive systematic search of records in MEDLINE by PubMed, Scopus, and EMBASE. We used the following general and Medical Subject Headings terms: “*tuberculosis, meningeal*,” *tubercular meningitides*, *TB meningitis*, *meningeal tuberculosis*, or *meningeal tuberculoses* occurring with *stroke*, *cerebrovascular disease*, *cerebral infarction*, *brain infarction*, *cerebral hemorrhage*, *brain hemorrhage*, or *vasculitis*. The literature search was limited to studies published from the inception of records in these major databases until July 2020.

### Data Collection

Two investigators (M.C.S. and A.I.E.) independently extracted data from the eligible studies and any discrepancies were resolved by both investigators. The following data were extracted from the relevant studies: author and year, publication date, study design, setting or geographic region, the number of included patients, age, sex, clinical manifestations, imaging findings, type of stroke, area of stroke, vascular territory affected, and outcomes. We also obtained information on the TBM stroke zones as defined by Hsieh et al,^9^ namely the TB zones and the ischemic zones. The TB zone is the area supplied by the medial lenticulostriate, thalamotuberal, and thalamoperforating arteries affecting the head of the caudate nucleus, genu, and anterior limb of internal capsule and anteromedial thalamus regions, while the ischemic zone is supplied by the lateral lenticulostriate, anterior choroidal, and thalamogeniculate artery affecting the lentiform nucleus, posterolateral thalamus, and posterior limb of internal capsule areas.^9^

### Statistical Analysis

We used frequencies and proportions for categorical variables and means (SD) or median (range) for continuous variables to summarize the data. Moreover, the 95% CIs for the population mean were computed when more than 1 study was available. Statistical analysis was performed using SPSS Statistics software for Macintosh, Version 24 (IBM Corporation). A value of *P* < .05 was considered statistically significant.

## Results

### Study and Population Characteristics

All included studies involved patients diagnosed with TBM who developed stroke in the course of their illness. The included articles consisted of 42 retrospective studies,^9,10,11,12,13,14,15,16,17,18,19,20,21,22,23,24,25,26,27,28,29,30,31,32,33,34,35,36,37,38,39,40,41,42,43,44,45,46,47,48,49,50^ 25 prospective studies^5,51,52,53,54,55,56,57,58,59,60,61,62,63,64,65,66,67,68,69,70,71,72,73,74^ and 4 randomized controlled trials^75,76,77,78^ with a total of 2194 patients with stroke among 8460 patients with TBM. For the demographics, the age of the patients ranged from 2 months to 85 years, with an overall women-to-men ratio of 0.6:1.

### Included Studies

A total of 852 journal articles (MEDLINE by PubMed, 343; Scopus, 471; Embase, 38) were identified using the search strategy. After duplicates were removed, 617 titles and abstracts were screened. After exclusion of 498 articles that did not fulfill the screening criteria, 119 full-text reports were assessed for eligibility. Forty-eight articles were subsequently excluded (ie, articles not in English, case reports or series, literature reviews, and articles with no report of stroke). We included a total of 71 studies in the final analysis (eFigure in the Supplement). Nearly all included patients who reported a stroke had cerebral infarction (2192 patients [99.9%]). Only 2 patients had intracerebral hemorrhage among patients with stroke (2 [0.1%]). The sample size for each study ranged from 17 to 806 patients (Table 1).

**Table 1.** Features of the Included Studies and Population

Study	Duration, y	Setting	Study design	Sample, No.	Median (range)	Women:men ratio
Al-edrus et al,^10^ 2007	NNR	Malaysia	Retrospective cohort	42	34.4 (18-62)	0.35:1
Alarcon et al,^21^ 2011	15	Ecuador	Retrospective cohort	310	34.5^a^	0.57:1
Anderson et al,^32^ 2010	18	New Zealand	Retrospective cohort	104	26.8 (1-81)	0.41:1
Andronikou et al,^43^ 2006	4	South Africa	Retrospective cohort	130	3 (2 mo-13)	0.47:1
Anuradha et al,^51^ 2010	1	India	Prospective cohort	100	30 (14-84)	0.49:1
Azeemuddin et al,^46^ 2019	10	Pakistan	Retrospective cohort	559	42 (17-97)	0.47:1
Bandyopadhyay et al,^62^ 2009	3	India	Prospective cohort	82	36.1 (8.3)^a^	NR
Bhargava et al,^47^ 1982	NR	India	Retrospective cohort	60	NR	NR
Bullock et al,^48^ 1982	1	South Africa	Retrospective cohort	52	NR	NR
Cagatay et al,^49^ 2004	11	Turkey	Retrospective cohort	42	33.9 (13.2)^a^	0.5:1
Cantier et al,^50^ 2018	12	France	Retrospective cohort	90	43 (29-58)	0.6:1
Chan et al,^68^ 2005	6	Hong Kong	Prospective cohort	40	53 (18-85)	0.4:1
Chen et al,^69^ 2014	4	Taiwan	Prospective cohort	38	52.1 (19.8)^a^	0.52:1
Chou et al,^11^ 2009	10	Taiwan	Retrospective cohort	43	53.3 (20-88)	0.48:1
Ersöz et al,^12^ 2012	11	Turkey	Retrospective cohort	60	27.5 (14-62)	0.82:1
George et al,^13^ 2012	2	India	Retrospective cohort	98	43.6 (14.4)^a^	0.60:1
Gu et al,^14^ 2015	4	China	Retrospective cohort	156	32.9 (18.6)^a^	0.59:1
Haji et al,^70^ 2019	3	Pakistan	Prospective cohort	110	NR	NR
Helbok et al,^15^ 2006	6	Thailand	Retrospective cohort	43	29.7 (16)^a^	0.72:1
Hosoglu et al,^16^ 2002	12	Turkey	Retrospective cohort	434	33 (13-83)	0.76:1
Hsieh et al,^9^ 1992	5	Taiwan	Retrospective cohort	40	57.9 (38-74)	NR
Hsu et al,^17^ 2010	6	Taiwan	Retrospective cohort	108	54.9 (18.6)^a^	0.52:1
Jinkins et al,^18^ 1991	NR	Saudi Arabia	Retrospective cohort	80	(2-76)	0.86:1
Kalita et al,^71^ 2017	1	India	Prospective cohort	122	32 (4-82)	0.90:1
Kalita et al,^72^ 2009	NR	India	Prospective cohort	26	23 (11-75)	1.17:1
Karande et al,^72^ 2005	3	India	Prospective cohort	123	72 (58.5)^a^	0.89:1
Karstaedt et al,^19^ 1998	3	South Africa	Retrospective cohort	56	33.9 (18-59)	0.66:1
Kingsley et al,^74^ 1987	1	United Kingdom	Prospective cohort	25	(1-70)	Not reported
Koh et al,^52^ 2006	5	Korea	Prospective cohort	38	34 (16-77)	1.24:1
Lan et al,^20^ 2001	5	Taiwan	Retrospective cohort	36	55 (16-83)	1.6:1
Leiguarda et al,^53^ 1988	NR	Argentina	Prospective cohort	65	NR	NR
Li et al,^22^ 2017	3	China	Retrospective cohort	154	41 (24-59)	0.77:1
Lu et al,^54^ 2015	7	China	Prospective cohort	36	47 (16-83)	0.56:1
Lu et al,^23^ 2001	5	Taiwan	Retrospective cohort	101	36.7 (14-81)	0.71:1
Mai et al,^75^ 2017	2	Vietnam	Randomized controlled trial	120	40 (31-53)	0.52:1
Merkler et al,^24^ 2017	8	US	Retrospective cohort	806	50 (17.1)^a^	0.59:1
Misra et al,^25^ 2000	3	India	Randomized controlled trial	17	18.8 (5-62)	0.31:1
Misra et al,^76^ 2010	NR	India	Retrospective cohort	118	30 (6-82)	1.03:1
Modi et al,^55^ 2017	5	India	Prospective cohort	209	30.4 (13.8)^a^	1:1
More et al,^56^ 2017	2	India	Prospective cohort	115	34.86 (16.57)^a^	0.89:1
Morgado et al,^26^ 2013	6	South Africa	Retrospective cohort	22	31 (19-57)	0.83:1
Napoli et al,^27^ 2019	7	Italy	Retrospective cohort	69	51.6 (16.9)^a^	0.35:1
Omar et al,^28^ 2011	2	South Africa	Retrospective cohort	34	3.5 (3 mo-15)	1.62:1
Ozates et al,^29^ 2000	8	Turkey	Retrospective cohort	289	29.9 (12.7)^a^	0.84:1
Pasticci et al,^30^ 2013	39	Italy	Retrospective cohort	30	NR	NR
Pienaar et al,^31^ 2009	NR	South Africa	Retrospective cohort	30	4.7 (1-13)	1.14:1
Qu et al,^33^ 2016	7	China	Retrospective cohort	105	NR	0.84:1
Raut et al,^57^ 2013	2	India	Prospective cohort	80	30.1 (14-68)	0.86:1
Roca et al,^34^ 2008	15	Spain	Retrospective cohort	29	34 (17-78)	0.41:1
Rohlwink et al,^58^ 2016	3	South Africa	Prospective cohort	44	3.3 (3 mo-13)	0.57:1
Rojas-Echeverri et al,^59^ 1996	3	Mexico	Prospective cohort	24	37 (18-65)	0.5:1
Samuel et al,^60^ 1987	2	India	Prospective cohort	127	(7 mo-5)	NR
Shah et al,^77^ 2014	NR	India	Prospective cohort	63	NR	1.25:1
Sharma et al,^35^ 2011	10	India	Prospective cohort	158	31.95 (13.96)^a^	0.74:1
Sharma et al,^61^ 2017	2	India	Prospective cohort	146	31.8 (15.01)^a^	0.46:1
Sheu et al,^36^ 2010	4	Taiwan	Prospective cohort	91	53.2 (19.8)^a^	0.64:1
Shoeman et al,^64^ 1988	2	South Africa	Retrospective cohort	198	NR	0.96:1
Shoeman et al,^63^ 1995	8	South Africa	Retrospective cohort	27	3.5 (1-7)	NR
Shukla et al,^65^ 2008	2	India	Prospective cohort	30	28.7 (14-56)	NR
Singh et al,^66^ 2012	2	India	Prospective cohort	47	28 (12-65)	0.96:1
Soni et al,^37^ 2019	4	India	Retrospective cohort	90	32 (10-82)	1.14:1
Springer et al,^38^ 2009	5	South Africa	Retrospective cohort	118	31.8 (18.3)^a^	NR
Sutlas et al,^39^ 2003	12	Turkey	Retrospective cohort	61	34.5 (16-74)	0.56:1
Synmon et al,^67^ 2016	2	India	Prospective cohort	93	32.3 (17.05)^a^	0.62:1
Tai et al,^40^ 2016	5	Malaysia	Retrospective cohort	51	35.1 (12.9)^a^	0.7:1
Teoh et al,^41^ 1989	NR	Hong Kong	Retrospective cohort	64	27.4 (21.7)^a^	0.68:1
Thwaites et al,^78^ 2015	2	Vietnam	Randomized controlled trial	27	31 (15-66)	NR
van Well et al,^42^ 2009	20	Netherlands	Retrospective cohort	554	2 (2-18)	0.91:1
Wasay et al,^44^ 2019	11	Pakistan	Retrospective cohort	559	NR	NR
Yasar et al,^45^ 2010	11	Turkey	Retrospective cohort	160	32.18 (13.62)^a^	1:1
Zhang et al,^5^ 2019	3	China	Prospective cohort	52	30.3 (9.9)^a^	0.36:1

Abbreviation: NR, not reported.

^a^ Mean value with SD (if available).

### Global Estimate of Proportions of Stroke in TBM

**Figure 1.**
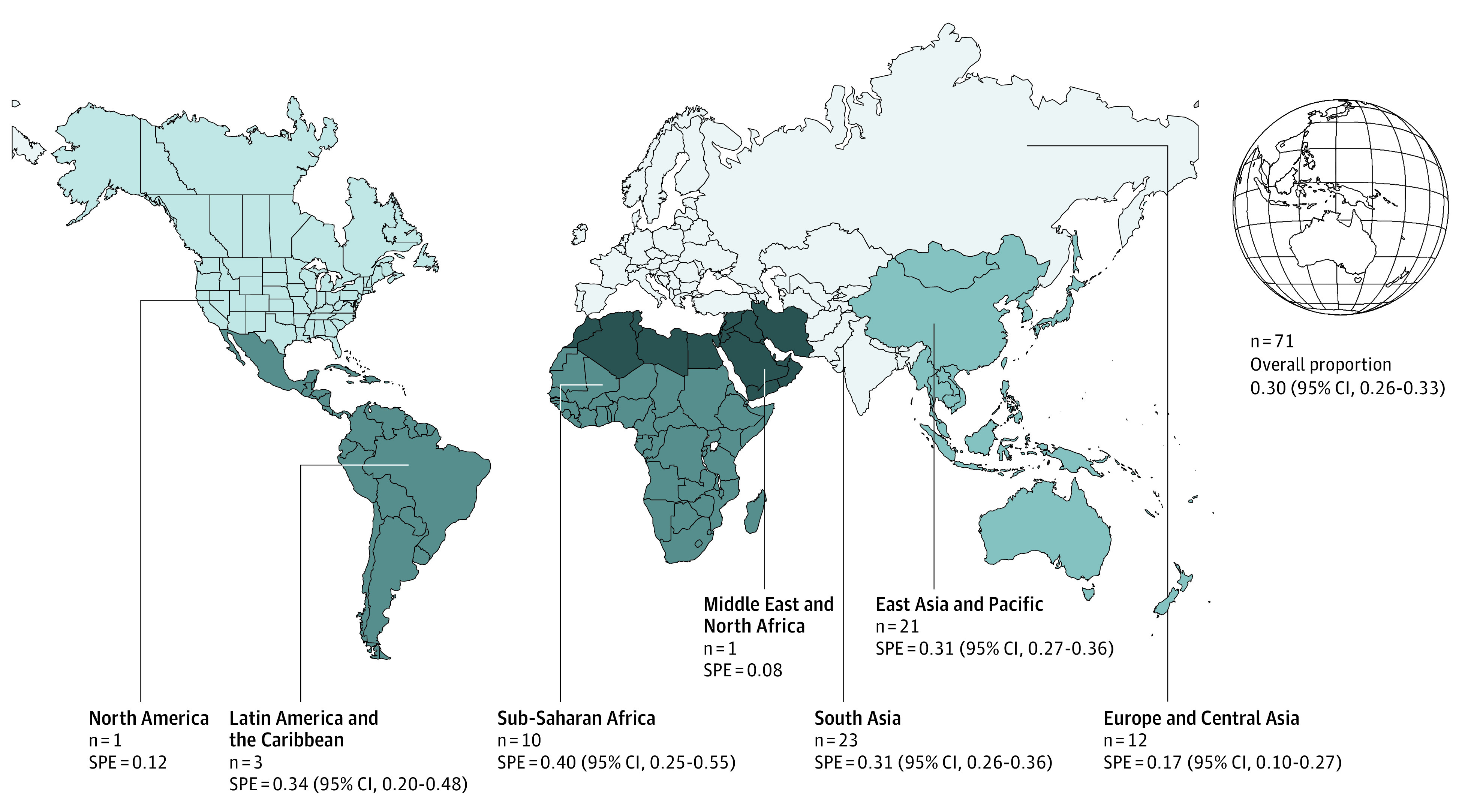
Regional Proportions of Stroke in Tuberculous Meningitis The map shows the number of included studies and proportions of stroke in each region and overall. SPE indicates stroke point estimate.

Across all 71 studies including 2194 patients with stroke and 8460 total patients with TBM, the frequency of stroke in TBM showed an overall point estimate of 0.26 (95% CI, 0.26-0.33) (Table 2). The pooled regional proportions of stroke among total patients with TBM ranged from a point estimate of 0.08 to 0.40 (Figure 1). In the East Asia and Pacific region, the point estimate was 0.31 (95% CI, 0.27-0.36) across 21 studies, with 455 patients with stroke and 1489 total patients with TBM (Figure 1). For Europe and Central Asia, the point estimate was 0.17 (95% CI, 0.10-0.27) in 12 studies, including 338 patients with stroke and 1843 total patients. The Latin America and the Caribbean region had a pooled point estimate of 0.34 (95% CI, 0.20-0.48) in 3 studies with 108 patients with stroke and 399 total patients. The sub-Saharan Africa region had an estimate of 0.40 (95% CI, 0.25-0.55) across 10 studies, with 274 patients with stroke and 711 total patients. The point estimate for the South Asia region was 0.31 (95% CI, 0.26-0.36) across 23 studies with 918 patients with stroke and 3132 total patients. In a single study (including 6 patients with stroke and 80 total patients), the Middle East and North Africa region had a point estimate of 0.12. There was also a single study of the North America region, with a point estimate of 0.08 across 95 patients with stroke and 806 total patients. The country-specific proportions of stroke in patients with TBM varied across the countries included (Table 2).

**Table 2.** Summary Estimates of Stroke in Tuberculous Meningitis per Country

Region/country	No. of studies	Patients with stroke, No.	Total patients, No.	Point estimate (95% CI)
Middle East and North Africa				
Saudi Arabia	1	6	80	0.08
North America				
US	1	95	806	0.12
Latin America and the Caribbean				
Argentina	1	25	65	0.38
Ecuador	1	72	310	0.23
Mexico	1	11	24	0.46
East Asia and Pacific				
China	5	154	568	0.27 (0.22-0.31)
Hong Kong	2	29	104	0.28 (0.19-0.36)
Korea	1	8	38	0.21
Malaysia	2	46	93	0.46 (0.10-0.85)
New Zealand	1	34	104	0.33
Vietnam	2	51	147	0.35 (0.27-0.42)
Europe and Central Asia				
France	1	50	90	0.56
Italy	2	17	99	0.17 (0.10-0.24)
Netherlands	1	164	554	0.30
Spain	1	4	29	0.14
Turkey	6	91	1046	0.09 (0.05-0.15)
United Kingdom	1	12	25	0.48
South Asia				
India	20	567	1904	0.32 (0.25-0.38)
Pakistan	3	351	1228	0.29 (0.25-0.33)
Taiwan	7	117	392	0.31 (0.21-0.42)
Thailand	1	16	43	0.37
Sub-Saharan Africa				
South Africa	10	274	711	0.40 (0.25-0.55)
Total	71	2194	8460	0.30 (0.26-0.33)

### Overall Clinical Manifestations of Stroke in TBM

The frequency for each clinical manifestation reported in patients with stroke in TBM ranged from 0.26 to 0.86 (eTable 1 in the Supplement). The point estimate was 0.69 (95% CI, 0.58-0.81) for altered sensorium in 8 studies with 126 cases among 189 total patients with TBM experiencing stroke; 0.42 (95% CI, 0.12-0.73) for cranial nerve palsy in 5 studies including 43 cases among 107 total patients; 0.86 (95% CI, 0.68-0.94) for fever across 7 studies including 226 cases among 302 total patients; 0.62 (95% CI, 0.41-0.84) for focal weakness or hemiplegia in 10 studies including 167 cases among 288 total patients; 0.76 (95% CI, 0.62-0.90) for headache across 6 studies including 119 cases among 158 total patients; 0.74 (95% CI, 0.61-0.87) for neck stiffness across 3 studies including 52 cases among 71 total patients; and 0.26 (95% CI, 0.13-0.45) for seizure across 7 studies including 62 cases among 290 total patients.

### Vascular Territories and Affected Areas of Stroke in TBM

**Figure 2.**
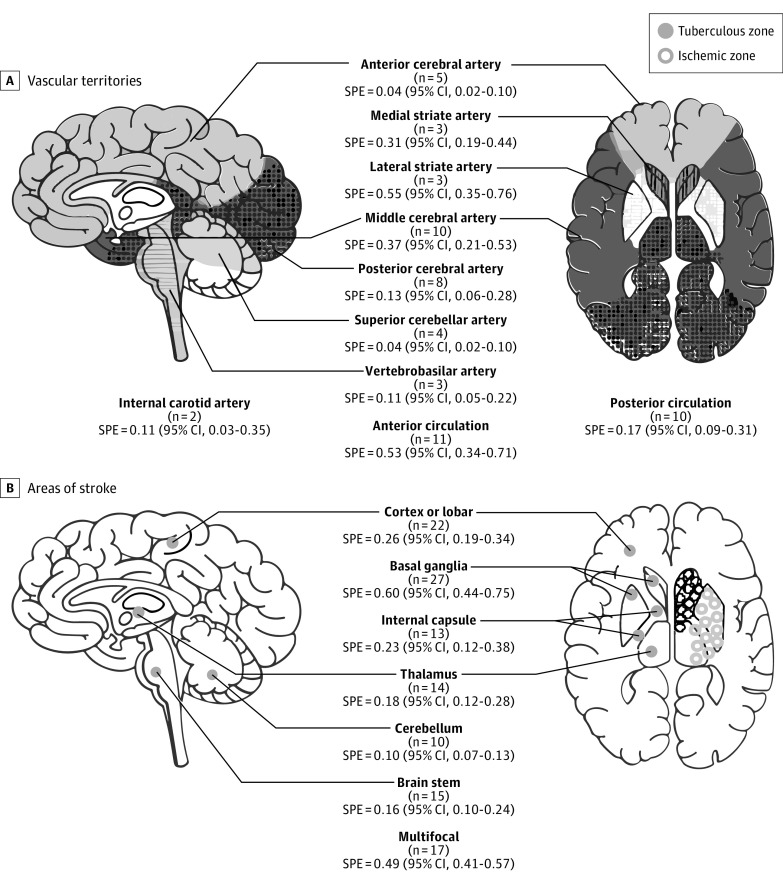
Studies and Summary Estimates of Vascular Territories and Areas of Stroke in Tuberculous Meningitis The figure shows the number of studies included in the analysis and frequency of stroke for various vascular territories and areas. SPE indicates stroke point estimate.

The frequency of vascular territories involved were the following: 0.04 (95% CI, 0.02-0.10) anterior cerebral artery across 5 studies including 5 cases among 151 total patients with TBM experiencing stroke; 0.11 (95% CI, 0.03-0.35) for internal carotid artery across 2 studies including 10 cases among 77 total patients; 0.37 (95% CI, 0.21-0.53) for middle cerebral artery across 10 studies including 82 cases among 229 patients; 0.13 (95% CI, 0.06-0.28) for posterior cerebral artery across 8 studies including 31 cases among 226 total patients; 0.55 (95% CI, 0.35-0.76) for lateral striate artery across 3 studies including 55 cases among 97 total patients; 0.31 (95% CI, 0.19-0.44) for medial striate artery across 3 studies including 30 cases among 97 total patients; 0.04 (95% CI, 0.02-0.10) for superior cerebellar artery across 4 studies including 4 cases among 116 total patients; 0.11 (95% CI, 0.05-0.22) for vertebrobasilar artery across 3 studies including 7 cases among 62 total patients; 0.53 (95% CI, 0.34-0.71) for anterior circulation across 11 studies including 145 cases among 304 total patients; and 0.17 (95% CI, 0.09-0.31) for posterior circulation across 10 studies including 57 cases among 287 total patients (Figure 2A). The summary estimates for areas affected by the stroke were as follows: 0.60 (95% CI, 0.44-0.75) in the basal ganglia across 27 studies including 551 cases among 1060 total patients; 0.23 (95% CI, 0.12-0.38) in the internal capsule across 13 studies including 134 cases among 524 total patients; 0.18 (95% CI, 0.12-0.28) in the thalamus across 14 studies including 120 cases among 651 total patients; 0.26 (95% CI, 0.19-0.24) in the cortex and lobar across 22 studies including 324 cases among 963 total patients; 0.16 (95% CI, 0.10-0.24) in the brainstem across 15 studies including 137 cases among 763 total patients; 0.10 (95% CI, 0.07-0.13) in the cerebellum across 10 studies including 39 cases among 435 total patients; and 0.49 (95% CI, 0.41-0.57) in multifocal areas across 17 studies including 373 cases among 707 total patients (Figure 2B).

### Outcomes of Patients With Stroke in TBM

The overall estimated proportions of mortality from patients with stroke in TBM were 0.22 (95% CI, 0.16-0.29) across 15 studies including 114 cases among 473 total patients (eTable 2 in the Supplement). The proportion of poor outcomes was 0.51 (95% CI, 0.37-0.66) across 10 studies including 141 cases among 284 total patients.

## Discussion

To the best of our knowledge, our review presents the most comprehensive evaluation of stroke in TBM with relevant information from 71 studies involving 2194 patients. This review provides extensive analyses of proportions of stroke across all regions as well as the clinical manifestations, type of stroke, area of stroke, vascular territory affected, and outcomes associated with this condition.

The consolidated evidence showed that the global frequency of stroke in TBM is approximately 30%, ie, 3 out of 10 patients with TBM may eventually develop a stroke. It is one of the most common complications of TBM patients across all age groups. Other complications of TBM include hydrocephalus (60%) followed by tuberculoma (10%), myelitis (10%), and seizure (10%).^70^ The previous reports by Misra et al^6^ described that approximately 13% to 57% of TBM patients had cerebral ischemia.^25^ Of note, nearly all patients who had stroke in the included studies developed cerebral infarction and only 2 patients had intracerebral hemorrhage. Several pathomechanisms were suggested in the development of this complication, including arteritis, vasospasm, arterial thrombosis, and compression of arteries by basal inflammatory exudate.^24^

Interestingly, TB was found to be widespread in resource-constrained areas. The global burden is estimated at 8.8 million incident cases, higher in Africa followed by Asia and Latin America.^79^ In our study, the top regions with the highest estimate of proportions of stroke in TBM were in sub-Saharan Africa (40%), Latin America and the Caribbean (34%), South Asia (31%), and East Asia and Pacific (31%). A review of stroke specifically in the South Asian population reported the conventional vascular risk factors to be highly prevalent, but infectious cause was also a significant contributor.^80^ Only 1 study was found in North America and the Middle East and North Africa. These are also regions which had a low incidence of TB according to the global tuberculosis report of 2019.^79^ The variation in numbers can be explained by regional differences in treatment practices, income, government policies, and the local effect of large countries like South Africa, where poor quality treatment extends the duration of the disease.^30,45^ On the other hand, we surmise that increased access to effective treatment leads to relatively lower frequency of stroke in TBM for the North American region. The top countries with the highest frequencies of stroke in TBM were Malaysia, South Africa, Vietnam, and India. These were considered middle-to-low–income countries that have a high burden of TB and limited access to health care.^42^

Stroke in TBM patients may be asymptomatic or present as silent stroke.^5^ Fever and headache are known to be the most common symptoms in TBM.^5^ Altered consciousness has been reported about 17% to 69% in patients with TBM, which was 69% in our pooled estimate.^5,30^ Moreover, patients with TBM experiencing stroke were more likely to present with focal weakness, which was found in 62% of patients in our analysis.^20,26,40^ Cranial nerve palsy is also an important symptom in patients with TBM, which may be due to increased intracranial pressure or exudates in the basilar region of the brain.^24^ Some studies have reported that these basilar exudates may be the culprit for stroke in the vulnerable areas of the brain, specifically the anteromedial thalamus, caudate nucleus, and anterior horn of the internal capsule.^5,9^ These structures make up the tubercular zone as described by Hsieh et al,^9^ which are supplied by the medial lenticulostriate, thalamotuberal, and thalamoperforating arteries. Cerebral ischemia have a predilection to develop in these areas and may subsequently aid clinicians in identifying the cause of stroke. Among the involved vascular territories, the most commonly affected by TB are those from the anterior circulation specifically the lateral striate artery, medial striate artery and middle cerebral artery. This corresponds to the brain region usually affected by stroke, specifically the basal ganglia, and internal capsule, which is consistent with our analysis.

In our analysis, we found pooled mortality and poor outcomes of 22% and 51% among patients with TBM experiencing stroke, respectively. In order to achieve better outcomes, early initiation of appropriate therapy is crucial. The current treatment guidelines recommend a first-line regimen for 2 months of isoniazid, rifampicin, pyrazinamide, and ethambutol followed by 10 months of isoniazid and rifampicin.^75^ Therapeutic interventions to reduce the frequency of stroke in patients with TBM have included steroids and antithrombotic therapy.^77^ It has been concluded in studies that the safety and efficacy of aspirin use needs further investigation by including a larger sample size and evaluating the long term survival and disability.^75,76^ Therefore, more research should be conducted to prevent stroke in this set of population to improve their clinical outcomes.

Our study provides a large data set of patients with TBM and stroke. We have reported extensive data that describes the overall, regional, and country-specific differences in the proportions of stroke in TBM. Due to the considerable number of stroke cases in the setting of TBM, further research on the interventions that aim to reduce the frequency of stroke are needed. We also recommend the evaluation of possible demographic and clinical factors associated with stroke in the TBM population, such as age, sex, duration and severity of disease, and the role of other comorbidities known to be associated with stroke such as hypertension, diabetes, dyslipidemia, and HIV. Finally, robust prognostic studies that evaluate the factors of outcomes in this population are warranted to guide clinical interventions.

### Limitations

This study had several limitations. The first was the heterogeneity of several pooled estimates. Another limitation was the exclusion of unpublished studies and non-English publications. In terms of clinical parameters, the population included were from varying age groups, including both adult and pediatric population, sex, and ethnicity. These parameters were difficult to assess because most studies reported pooled data. Consequently, different imaging modalities were used to diagnose stroke such as cranial CT scan, MRI, and angiography. The timing of stroke in TBM and the reported outcomes varied across the studies. Retrospective studies are known to have an inherent recording bias that can obscure the validity of the estimates.

## Conclusions

In this systematic review, stroke was considerably prevalent among patients with TBM, ie, 3 out of 10 patients with TBM would be estimated to develop stroke in the course of their illness based on these data. The common clinical presentations of TBM with stroke were fever, headache, neck stiffness, altered sensorium, focal weakness or hemiplegia, cranial nerve palsy, and seizure. Nearly all reported cases were cerebral infarctions; lateral striate, middle cerebral and medial striate arteries were commonly affected while basal ganglia, cortex or lobar, and internal capsule were the frequently injured areas. Based on these results, approximately 5 out of 10 patients would be estimated to have poor outcomes while 2 out of 10 may expire. Global trends of stroke occurrence in TBM may have regional or country-specific characteristics that may influence disability and mortality in these patients.

## Notes

Supplement. **eTable 1.** Summary Estimates of Clinical Manifestations of Stroke in Tuberculous Meningitis **eTable 2.** Pooled Estimates of Outcomes of Stroke in Tuberculous Meningitis **eFigure.** PRISMA Information Diagram for the Inclusion of Relevant Articles **eAppendix.** Search Strategy
