# Supplementary material for: Global Frequency and Clinical Features of Stroke in Patients With Tuberculous Meningitis: A Systematic Review
Source: JAMA Netw Open. 2022 Sep 1;5(9):e2229282. doi: 10.1001/jamanetworkopen.2022.29282 (PMC9437750; doi:10.1001/jamanetworkopen.2022.29282)
Supplement: Supplement. — eTable 1. Summary Estimates of Clinical Manifestations of Stroke in Tuberculous Meningitis eTable 2. Pooled Estimates of Outcomes of Stroke in Tuberculous Meningitis eFigure. PRISMA Information Diagram for the Inclusion of Relevant Articles eAppendix. Search Strategy [file jamanetwopen-e2229282-s001.pdf]

Supplemental Online Content

Sy MCC, Espiritu AI, Pascual JLR. Global frequency and clinical features of stroke in tuberculous meningitis. *JAMA Netw Open.* 2022;5(9):e2229282.
doi:10.1001/jamanetworkopen.2022.29282

eTable 1. Summary estimates of clinical manifestations of stroke in tuberculous meningitis

eTable 2. Pooled estimates of outcomes of stroke in tuberculous meningitis

eFigure. PRISMA information diagram for the inclusion of relevant articles

eAppendix. Search strategy

This supplemental material has been provided by the authors to give readers additional information about their work.

© 2022 Sy MCC et al. *JAMA Network Open.*

eTable 1. Summary estimates of clinical manifestations of stroke in tuberculous meningitis

Clinical manifestations	Number of studies	Patients with specified clinical manifestation	Total patients	Point estimate [95% confidence interval]
Altered sensorium	8	126	189	0.69 [0.58-0.81]
Cranial nerve palsy	5	43	107	0.42 [0.12-0.73]
Fever	7	226	302	0.86 [0.68-0.94]
Focal weakness/ Hemiplegia	10	167	288	0.62 [0.41-0.84]
Headache	6	119	158	0.76 [0.62-0.90]
Neck stiffness	3	52	71	0.74 [0.61-0.87]
Seizure	7	62	290	0.26 [0.13-0.45]

© 2022 Sy MCC et al. *JAMA Network Open.*

eTable 2. Pooled estimates of outcomes of stroke in tuberculous meningitis

Outcomes	Number of studies	Patients with reported outcomes	Total patients analyzed	Overall estimate
Mortality	15	114	473	0.22 [0.16-0.29]
Poor outcomes	10	151	284	0.51 [0.37-0.66]
Recovered	15	359	473	0.78 [0.71-0.84]

© 2022 Sy MCC et al. *JAMA Network Open.*

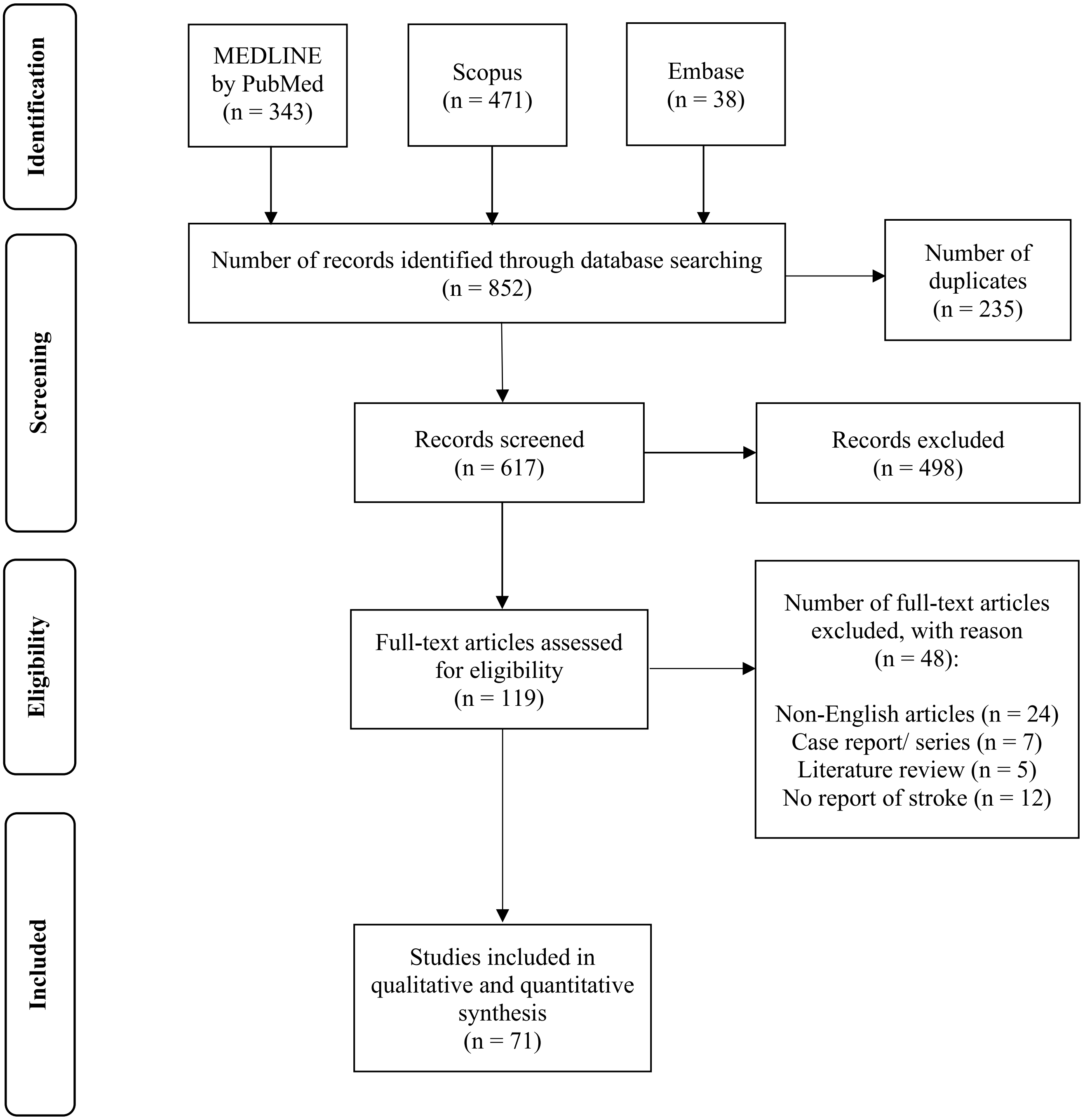

eFigure. PRISMA information diagram for the inclusion of relevant articles

© 2022 Sy MCC et al. *JAMA Network Open.*

eAppendix. Search strategy

- **MEDLINE by PubMed**

#1: Tuberculosis, meningeal OR Tubercular meningitides OR TB Meningitis OR Meningeal tuberculosis
#2: Meningeal tuberculoses OR Meningeal tuberculosis
#3: {OR #1-#2}
#4: Stroke OR Cerebrovascular disease OR Cerebral infarction OR Brain infarction
#5: Cerebral hemorrhage OR Brain hemorrhage
#6: Vasculitis OR Vasculitides
#7: {OR #5-#6-#7}
#8: {AND #3,#7}

- **Scopus**

#1: Tuberculosis, meningeal OR Tubercular meningitides OR TB Meningitis OR Meningeal tuberculosis
#2: Meningeal tuberculoses OR Meningeal tuberculosis
#3: (#1 OR #2)
#4: Stroke OR Cerebrovascular disease OR Cerebral infarction OR Brain infarction
#5: Cerebral hemorrhage OR Brain hemorrhage
#6: Vasculitis OR Vasculitides
#7: (#4 OR #5 OR #6)
#19: #3 AND #7

- **EMBASE**

((Tuberculosis, meningeal OR Tubercular meningitides OR TB Meningitis OR Meningeal tuberculosis OR Meningeal tuberculoses OR Meningeal tuberculosis) AND (Stroke OR Cerebrovascular disease OR Cerebral infarction OR Brain infarction OR Vasculitis OR Vasculitides))

© 2022 Sy MCC et al. *JAMA Network Open.*
